# P-334. Oral HIV Pre-exposure prophylaxis (PrEP) in the Commonwealth of the Bahamas: 2016-2023. A Comprehensive Review of Demographics, Clinical features, Roll out and Implementation, Uptake and Continuation

**DOI:** 10.1093/ofid/ofaf695.553

**Published:** 2026-01-11

**Authors:** Tavanette C Ingraham, Alphanso Blake, Glenise A Johnson, Stephen A Kemp, Clemon George, Nikkiah Forbes

**Affiliations:** Princess Margaret Hospital, Nassau, New Providence, Bahamas, The; University of the West Indies School of Clinical Medicine and Research, Nassau, TheBahamas, Nassau, New Providence, Bahamas, The; Ministry of Health & Wellness, TheBahamas, Nassau, New Providence, Bahamas, The; Ministry of Health and Wellness, TheBahamas, Nassau, New Providence, Bahamas, The; Buffalo State University, Buffalo, New York; Princess Margaret Hospital, Nassau, New Providence, Bahamas, The

## Abstract

**Background:**

Oral HIV PrEP was introduced in The Bahamas in June 2016, making The Bahamas the first country in the English-speaking Caribbean to include this prevention tool. These antiretroviral drugs, with efficacy of up to 96% against HIV, are offered without cost to clients. The aim of the study was to conduct a comprehensive review of the PrEP programme, to determine next steps to improve its effectiveness in reducing new HIV infections in The Bahamas.

**Methods:**

A comprehensive review of the National PrEP programme (June 2016-December 2023) was done. All participants enrolled between June 2016 to December 2023 were eligible for this retrospective review. De-identified data were abstracted into REDCap and confidentiality maintained. Data were analyzed using Stata 16 and reported as means and standard deviation, counts and percentages where necessary. Key variables included client's uptake, continuation of PrEP, clinical and demographic information, risk profile, STI's status, seroconversion and PrEP side effects. This was a minimal risk study and approval obtained from the Public Hospital Authority/UWI and Ministry of Health Ethics Committees.

**Results:**

Of the 325 clients enrolled, 76.4% were males and 55.6% were between 25-44 years. Serodiscordant couples and Men who had sex with men accounted for 45% and 40.8% respectively. Enrolment increased each year and 99% of clients began the PrEP programme. Continuation on PrEP was 50.7%. Prior to initiating PrEP, 5.8% tested positive for an STI and 4% tested positive for an STI while on PrEP; syphilis being the most common STI at 2.7%. New HIV seroconversion among those reporting suboptimal PrEP adherence was 3%. Inconsistent condom use was reported at 66.1%. Side effects reported were gastrointestinal at 2.8%.

**Conclusion:**

A steady increase in enrolment over time suggests increased awareness of the programme. While PrEP uptake was good, continuation was suboptimal. Further, a 3% seroconversion rate suggests an ongoing need to counsel clients on the need to be adherent, or to increase condom use during drug holidays. However, there is a need for further studies to determine the reasons for PrEP discontinuation and condom use failure.

**Disclosures:**

All Authors: No reported disclosures

